# Aqueous humour concentrations after topical apPlication of combinEd levofloxacin-dexamethasone eye dRops and of its single components: a randoMised, assEssor-blinded, parallel-group study in patients undergoing cataract surgery: the iPERME study

**DOI:** 10.1007/s00228-020-02863-7

**Published:** 2020-04-13

**Authors:** Michele Figus, Chiara Posarelli, Dario Romano, Marco Nardi, Luca Rossetti

**Affiliations:** 1 Department of Surgical, Medical, Molecular Pathology and of Critical Area, University of Pisa, Via Paradisa, 2, 56100 Pisa, Italy; 2 Eye Clinic, ASST Santi Paolo e Carlo, University of Milan, Milan, Italy

**Keywords:** Levofloxacin, Dexamethasone, Combination eye drop, Aqueous humour

## Abstract

**Purpose:**

To evaluate the penetration of levofloxacin and dexamethasone sodium phosphate into the aqueous humour (AH) after administration in combination and as single molecules. Evaluation of the penetration of those agents in the site of action and their pharmacodynamic potential activity in view of the intended clinical use after cataract surgery.

**Methods:**

Randomised, assessor-blinded, parallel-group. Patients scheduled for cataract surgery were assigned in a 1:1:1 ratio to: levofloxacin + dexamethasone sodium phosphate (L-DSP), Levofloxacin (L) or Dexamethasone sodium phosphate (DSP) eye drops. Either test or reference drugs were instilled in the cul-de-sac twice, 90 and 60 min before paracentesis.

**Results:**

A total of 125 patients completed the study. Fraction of dose absorbed in the anterior chamber was 3.8–4.2 · 10^−4^ for levofloxacin and 0.3–0.4 · 10^−4^ for dexamethasone, respectively. No notable differences in concentration of levofloxacin were found between L-DSP arm (1.970 nmol/ml) and L arm (2.151 nmol/ml). The concentrations of levofloxacin were well above the MICs for the most frequent Gram-positive and Gram-negative eye pathogens. Dexamethasone concentrations were slightly lower in L-DSP arm (0.030 nmol/ml) than in DSP arm (0.042 nmol/ml), but still in the pharmacodynamically active range in the site of action. The difference was not clinically relevant. DSP was not detected in any HA sample, suggesting its full hydrolysis to free dexamethasone.

**Conclusion:**

Our results confirm that no interaction is evident on the corneal penetration of levofloxacin and dexamethasone which reach pharmacologically active concentrations when instilled as fixed combination eye drops to patients undergoing cataract surgery.

**Trial registration:**

ClinicalTrials.gov Identifier: NCT03740659

**Electronic supplementary material:**

The online version of this article (10.1007/s00228-020-02863-7) contains supplementary material, which is available to authorized users.

## Introduction

Postoperative endophthalmitis is an inflammatory condition of the eye, due to an infectious process from bacteria, fungi or, on rare occasions, parasites that enter the eye during the perioperative period [1]. After an initial incubation phase lasting 16–18 h to a few days, an acceleration phase and a destructive phase of the infection develop. The acceleration phase follows primary infection of the posterior segment and leads to inflammation of the anterior chamber [1].

The prophylaxis of postoperative bacterial ocular infections such as endophthalmitis consists in the administration of intraocular, usually intracameral or subconjunctival antibiotics during surgery. Both pre- and postoperative topical applications are also used frequently [2]. To increase efficacy, a broad-spectrum antibiotic is indicated, and topical use of quinolones may represent a rational choice given their bactericidal activity against both the gram-positive and gram-negative bacteria most frequently responsible for bacterial eye infections [3]. Postoperative care after cataract surgery consists of topical anti-inflammatory and antibacterial drug prophylaxis.

Eye Drop Combi (L-DSP) is a fixed dose combination (FDC) of eye drops solution, with 0.5% levofloxacin and 0.132% dexamethasone sodium phosphate (corresponding to 0.1% free dexamethasone) as active ingredients, under development by NTC Srl, Milan (Italy).

The pharmacokinetics [4–9] of either levofloxacin or dexamethasone administered individually confirm that after application as an eye drop composition, either active drug reaches significant AH concentrations, with peaks of aqueous levels between 90 and 150 min, and detectable concentrations after 12 h from dosing. Animal studies confirmed that after eye instillation of L-DSP at the human doses and for a short period of time, no local or systemic toxicity is expected (NTC, data on file, 2019).

The primary objective of this study was to analyse the AH drug concentrations of levofloxacin and dexamethasone instilled in the cul-de-sac either in combination or as single components. Secondary objectives included the evaluation of the penetration of those agents in the site of action and their pharmacodynamic potential activity in view of the intended clinical use for postoperative prophylaxis in cataract surgery.

## Material and methods

### Design

This was a multicentre, randomised, assessor-blinded, parallel-group clinical study. Patients scheduled for cataract surgery from September 2018 until December 2018 were recruited at the Ophthalmic Unit both of Azienda Ospedaliero Universitaria Pisana (Centre 001) and at the Eye Clinic and Head and Neck Department, ASST Santi Paolo e Carlo Hospital, University of Milan (Centre 002).

Randomisation list, monitoring of clinical centres and data management and statistics were provided by OPIS, an EU independent, full service clinical Contract Research Organization (CRO) with HQ in Desio (MB, Italy). The randomisation list was generated at OPIS by a statistician not involved in the study through a validated SAS program (SAS® for Windows release 9.4 (64-bit)). The allocation ratio was 1:1:1, and the randomisation was managed by means of an electronic system (Clinical.NET).

The study was full GCP and GLP. All procedures performed in the study involving human participants were in accordance with the ethical standards and with the 1964 Helsinki declaration and its later amendments. The protocol was approved by the institutional research committees of the investigating centres, i.e. Comitato Etico Regione Toscana Area Vasta Nord Ovest c/o Azienda Ospedaliero Universitaria Pisana via Roma, 67 - 56126 Pisa (PI) (approval letter on May 2, 2018) and Comitato Etico Milano Area 1 c/o ASST FBF Sacco - P.O. L. Sacco Via G.B. Grassi, 7420157 Milano (approval letter on September 9, 2018).

### Patients

Informed consent was obtained from all patients for being included in the study. Main eligibility criteria included patients of both genders, aged ≥ 40 years, and scheduled for phacoemulsification. Corneal thickness measured by pachymetry was between 450 and 600 μm, and corneal integrity was confirmed by means of fluorescein test. Patients must have an adequate pupil dilation. Patients without confirmed corneal epithelium integrity, with glaucoma or with history of ocular surgery, or corneal disease or dystrophy, or ocular trauma with corneal damage, or acute ocular inflammation in the previous 6 months were excluded. Also excluded were female patients of childbearing potential without a negative pregnancy test, patients under treatment with an ophthalmic investigational drug in the 3 months prior to screening or treated within 12 h before start of cataract surgery with any topical ocular drug other than study drugs and instillation of topical anaesthetic (oxybuprocaine hydrochloride) followed by povidone-iodine within 10 min before start of surgery, i.e. at least 80 and 50 min after the first and second drop, respectively). Finally, those patients treated with any topical or systemic steroid or antibiotic drug in the 7 days prior to cataract surgery were excluded, too.

At screening, which must take place not more than 28 days before planned surgery, information was collected concerning demographics, medical history and any use of concomitant medications. Pachymetry and fluorescein test were performed to measure corneal thickness and to confirm corneal epithelium integrity. Prior to surgery, a pre-dose visit was done to confirm eligibility criteria.

### Treatments

**Fig. 1:**
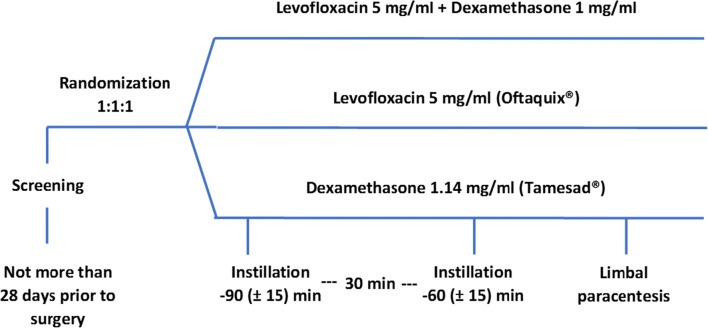
Study design

Eligible patients were randomly assigned to one of the three following treatment arms in a 1:1:1 ratio via an Interactive Web Response System (IWRS) provided by OPIS (Fig. 1):

- Test drug: levofloxacin 5 mg/ml + dexamethasone sodium phosphate 1.32 mg/ml eye drops (L-DSP)^1^ two 30-μl doses, 30 min apart, one 90 ± 15 min prior to surgery and the other 60 ± 15 min prior to surgery. Total dose instilled: levofloxacin 300.0 μg (= 830.17 nMoles) and dexamethasone sodium phosphate 79.2 μg (= 153.37 nMoles).
- Reference A: levofloxacin 5 mg/ml eye drops (Oftaquix®): two 30-μl doses, 30 min apart, one 90 ± 15 min prior to surgery and the other 60 ± 15 min prior to surgery. Total dose instilled: levofloxacin 300.0 μg (= 830.17 nMoles)
- Reference B: dexamethasone 1.5 mg/ml eye drops (Tamesad®, DSP): two 26-μl doses, 30 min apart, one 90 ± 15 min prior to surgery and the other 60 ± 15 min prior to surgery. Total dose instilled: dexamethasone sodium phosphate 78 μg (= 151.04 nMoles)

### Procedure

Administration of study drugs was performed by qualified health care personnel using a micropipette and dispenser. Doses were administered at the external canthus while applying pressure at the internal canthus to prevent drainage of the study drug. Phacoemulsification and limbal paracentesis were performed by an experienced surgeon. Approximately 50 μl of AH was drawn from the anterior chamber with a 30-gauge needle syringe. After paracentesis, a solution of tropicamide + phenylephrine HCl + lidocaine HCl (Mydrane®) was intracamerally injected to obtain mydriasis and intraocular anaesthesia. Following surgery, patients were treated with antibiotics and/or steroids according to local clinical practice.

The AH was stored immediately in vials at − 80 °C and subsequently analysed in a central laboratory. Vial labels contained the study kit number but no information regarding the drug received. The laboratory analyst was blinded to the drug administered, and all samples were analysed for the concentration of three molecules (levofloxacin, dexamethasone sodium phosphate and dexamethasone) by means of a liquid chromatography tandem mass spectrometry method (LC-MS-MS) developed and validated by the Central Laboratory Ticinumlab (Novara, Italy). Details of the procedure are available in Online Resource 1.

### Analytical method

The analytes were AH concentrations of levofloxacin, dexamethasone sodium phosphate (DSP) and dexamethasone. A full validation of a specific liquid chromatography tandem mass spectrometry (LC-MS-MS) method quantifying levofloxacin, DSP and its metabolite dexamethasone in human aqueous humour was performed according to a predefined study protocol. Description of the method is available as Online Resource 1.

The analytical procedure was blinded in full GCLP conditions.

### Statistical analysis

Continuous data were summarised with standard descriptive statistics. Categorical data were summarised by frequencies and percentages.

Aqueous humour concentrations of levofloxacin and dexamethasone, measured by LC-MS-MS, were summarised by treatment group, and 95% confidence limits were provided.

By considering the average volume of the anterior chamber as 161.2 μl in > 50-year-old Caucasian patients as reported by Fan et al. 2019 [10], both the quantity of the analytes present in the anterior chamber at the time of sampling and the fraction of absorbed dose *F* were calculated. Those data were also summarised by treatment group, and 95% confidence limits were provided.

The analysis was carried out on the following populations:

- Per Protocol (PP): all patients who completed the study without any major protocol deviations. Subjects were analysed according to the study treatment they actually received.
- Full Analysis Set (FAS): all randomised patients. According to Intention to Treat (ITT) principles, all patients were analysed considering the randomised treatment assignment.
- Safety (SAF): all patients who received at least one dose of study treatment. Subjects were analysed according to the study treatment they actually received (data not shown).

No formal statistical hypothesis was formulated; all analyses were descriptive in nature, and no inferential statistical tests were planned.

All outputs were produced using SAS® release 9.4 (SAS Institute, Inc., Cary, NC, USA).

### Sample size

The study was to enrol 120 patients, 40 in each treatment group. The sample size was estimated based on the expected precision (width of the 95% confidence interval) of the estimates of the study drug concentrations:

#### Dexamethasone

When the sample size is 40, a two-sided 95% confidence interval for a single mean extends 4.648 ng/ml from the observed mean assuming a standard deviation of 15 ng/ml and a confidence interval based on large sample *z* statistic.

#### Levofloxacin

When the sample size is 40, a two-sided 95% confidence interval for a single mean extends 61.676 ng/ml from the observed mean assuming a standard deviation of 199.022 ng/ml and a confidence interval based on large sample *z* statistic.

## Results

### Patient disposition

Overall, 133 patients were screened and entered the study treatment between September and December 2018. One patient was instilled a mydriatic agent by error (non-fulfilment of exclusion criteria) and was rescreened in a different occasion for the other eye, making a total of 134 screened patients/eyes. One hundred thirty-one (131) patients (97.76%) passed screening and were enrolled. Three (3) patients (2.24%) were not enrolled: two were screening failures and one withdrew consent. Six out of the 131 enrolled patients were not randomised: two withdrew the consent; one had punctate keratitis; one had surgery postponed after study completion because a required ECG had not been performed; one, already reported, was instilled a mydriatic agent by mistake and was then rescreened for the other eye; finally, one was not randomised because a freezer for sample storage was not in working order at the due time.

One hundred twenty-five (125) patients (i.e. 95.42% of screened patients) were randomised: 42 (33.60%) to the levofloxacin + dexamethasone sodium phosphate arm (L-DSP), 42 (33.60%) to the levofloxacin arm (L) and 41 (32.80%) to the dexamethasone sodium phosphate arm (DSP). All randomised patients received Mydrane® and thus completed the study. Protocol deviations were reported for 9 patients. Three patients (2.4%) had been treated with systemic steroids in the 7 days prior to cataract surgery, and 3 received a different drug than the one assigned by randomisation. Two patients received the treatment out of the required time window, and one patient had a history of corneal disease. Those patients did not enter in the PPS.

### Demographics and baseline characteristics

Demographic characteristics of the patient population are detailed in Table 1. Patients were on average 74 years of age, 55.20% were female and all but three (97.60%) were Caucasian. No clinically relevant differences in demographic and background characteristics were found among groups. As expected for patients of this age, the most common ongoing medical conditions were vascular disorders (mainly hypertension: 57.6%) and metabolism and nutritional disorders (diabetes mellitus, 18.4%).

**Table 1:** Demographic characteristics of the patient population (FAS)

		L-DSP	L	DSP
		(*N* = 42)	(*N* = 42)	(*N* = 41)
Age (years)	Mean	72.45	75.38	74.59
	SD	7.60	8.38	8.00
	Range, min–max	55–88	48–87	57–88
Gender *N* (%)	Male	15 (35.7%)	21 (50%)	20 (48.8%)
	Female	27 (64.3%)	21 (50%)	21 (51.2%)
Ethnics	Caucasian	42 (100%)	40 (95.2%)	40 (97.6%)
	Black or African American	0	1 (2.4%)	0
	Asian	0	1 (2.4%)	1 (2.4%)
Pachymetry	Mean	531.83	526.50	527.29
	SD	31.26	30.84	34.55
	Range, min–max	455–595	451–581	460–596

Treatment groups: *L-DSP* levofloxacin + dexamethasone sodium phosphate, *L* levofloxacin, *DSP* dexamethasone sodium phosphate

With regard to ocular medical conditions and surgical history, 8.80% of patients had an ongoing ocular condition of the eye to be operated, namely 4.76% of the L-DSP group, 4.76% of the levofloxacin group and 17.07% of the DSP group. The most common conditions were exfoliation syndrome (*N* = 3, 2.4%) (2 patients in the L-DSP group and 1 in the levofloxacin group) and diabetic retinopathy (*N* = 2, 1.60%) (both in the DSP group).

All patients underwent pachymetry, and all presented corneal thickness within range. Mean corneal thickness was 531.83 ± 31.26 μm in the L-DPS group, 526.50 ± 30.84 μm in the levofloxacin group and 527.29 ± 34.55 in the DPS group, respectively. All eyes scheduled for surgery presented intact corneas at the fluorescein test (Oxford scheme grade 0).

### Analytical method validation

The analytical method was fully validated for selectivity, linearity, within and between precision, and accuracy. LLOQ was 0.014 nmol/ml for levofloxacin, 0.058 nmol/ml for DSP and 0.013 nmol/ml for dexamethasone. Matrix effect was excluded in blank matrix spiked with analytes. Matrix effect was also excluded in presence of oxybuprocaine, iodopovidone and benzalkonium chloride, the drugs co-administered with either test or references.

The validation details are available as Online Resource 2.

### AH concentrations

**Fig. 2:**
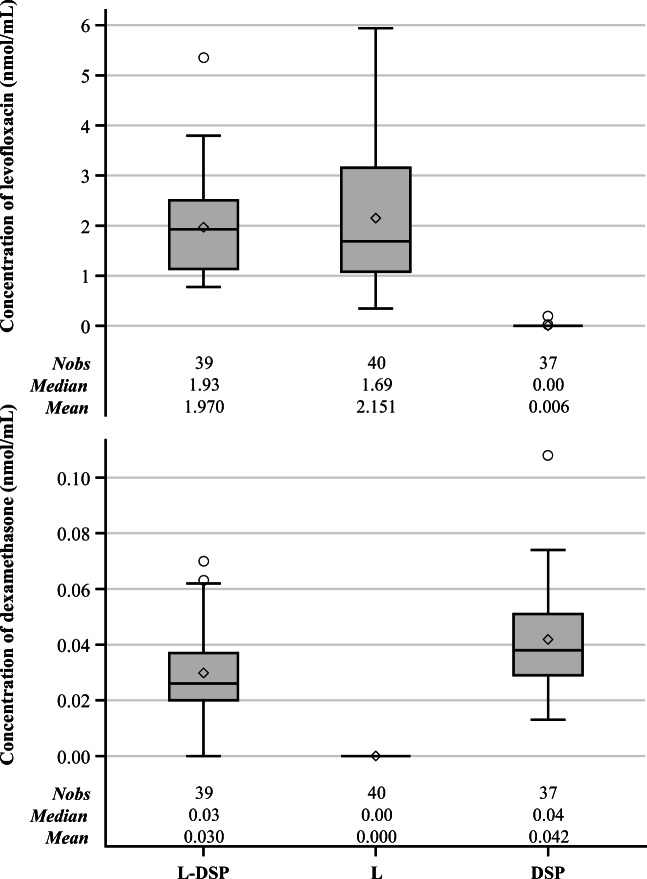
AH concentrations of levofloxacin (top) and of dexamethasone (bottom) after ocular instillation of levofloxacin + dexamethasone sodium phosphate (LDSP), levofloxacin (L) or dexamethasone sodium phosphate (DSP). See doses in the text. Data are means ± SD; Nobs = number of observations

The results of the aqueous humour analysis in the PPS were as follows (details in Table 2 and Fig. 2):

- Levofloxacin’s average concentration was 1.970 nmol/ml (95% CI (1.648; 2.292)) in the L-DSP group (corresponding to 711.899 ng/ml (95% CI 595.538; 828.260)) and 2.151 nmol/ml (95% CI (1.708; 2.594)) in the levofloxacin group (corresponding to 777.307 ng/ml (95% CI 617.220; 937.394)). Levofloxacin was also detected in two patients assigned to the DSP arm (0.014 nmol/ml and 0.193 nmol/ml respectively), but this could not be attributed to a specific cause. The samples were retested, and the one previously containing 0.014 nmol/ml (very near to LLOQ) no longer presented trace of levofloxacin.
- Dexamethasone average concentration was 0.030 nmol/ml (95% CI (0.025; 0.035)) in the L-DSP group (corresponding to 11.774 ng/ml (95% CI 9.812; 13.736)) and 0.042 nmol/ml (95% CI (0.035; 0.048)) in the DSP group (corresponding to 16.483 ng/ml (95% CI 13.736; 18.838)). Dexamethasone sodium phosphate was not detected in any AH sample, most likely due to its full hydrolysis to free dexamethasone.

**Table 2:** Aqueous humour concentrations (nmol/ml) - observed values (PPS)

	Concentration of levofloxacin (nmol/ml)			Concentration of dexamethasone (nmol/ml)		
	L-DSP	L	DSP	L-DSP	L	DSP
	(*N* = 39)	(*N* = 40)	(*N* = 37)	(*N* = 39)	(*N* = 40)	(*N* = 37)
Mean	1.970	2.151	0.006	0.030	0.000	0.042
SD	0.99	1.39	0.03	0.02	0.00	0.02
LL CI 95%	1.648	1.708	− 0.005	0.025	-	0.035
UL CI 95%	2.292	2.594	0.016	0.035	-	0.048
Min	0.777	0.347	0.000	0.000	0.000	0.013
Q1	1.14	1.08	0.00	0.02	0.00	0.03
Median	1.93	1.69	0.00	0.03	0.00	0.04
Q3	2.51	3.16	0.00	0.04	0.00	0.05
Max	5.361	5.942	0.193	0.070	0.000	0.108

Concentrations of dexamethasone sodium phosphate were always under the LLOQ and are not reported

Treatment groups: *L-DSP* = levofloxacin + dexamethasone sodium phosphate, *L* levofloxacin, *DSP* dexamethasone sodium phosphate. *LL* lower limit, *UL* upper limit, *CI* confidence interval

One vial containing aqueous humour was damaged and did not allow the analysis of AH drug concentrations for a patient allocated to the L-DSP group. This non-protocol deviation led to the patient’s exclusion from the per protocol set but not from the safety set.

### QC samples

The back-calculated concentrations in the in-house control samples were within ± 15% of the nominal value for all samples but one concentration of levofloxacin, which was + 20% of the nominal value (data not shown).

### Fraction *F* of dose absorbed in the anterior chamber

The fraction *F* of dose absorbed in the anterior chamber at the time of AH withdrawal is reported in Table 3. *F* of levofloxacin was 3.8 ± 1.9 · 10^−4^ after instillation of L-DSP and 4.2 ± 2.7 · 10^−4^ after instillation of L alone (between groups difference not significant). The *F* was lower for dexamethasone both after instillation of L-DSP (0.3 ± 0.2 · 10^−4^) and of DSP alone (0.4 ± 0.2 · 10^−4^).

**Table 3:** Fraction *F* of dose absorbed in the anterior chamber at the time of AH withdrawal (data · 10^−4^) - observed values (PPS)

	Dexamethasone *F*		Levofloxacin *F*	
	L-DSP	DSP	L-DSP	L
	(*N* = 39)	(*N* = 37)	(*N* = 39)	(*N* = 40)
Mean	0.3	0.4	3.8	4.2
SD	0.2	0.2	1.9	2.7
LL CI 95%	0.3	0.4	3.2	3.3
UL CI 95%	0.4	0.5	4.5	5.0
Min	0.0	0.1	1.5	0.7
Q1	0.2	0.3	2.2	2.1
Median	0.3	0.4	3.7	3.3
Q3	0.4	0.5	4.9	6.1
Max	0.7	1.2	10.4	11.5

Treatment groups: *L-DSP* levofloxacin + dexamethasone sodium phosphate, *L* levofloxacin, *DSP* dexamethasone sodium phosphate. *LL* lower limit, *UL* upper limit, *CI* confidence interval

### Ratio between MICs and AH levofloxacin concentrations

The mean concentration of levofloxacin in L-DSP group corresponded to 711.899 ng/ml (95% CI 595.538; 828.260). The aqueous humour concentrations were compared with the minimum inhibitory concentrations (MIC) reported by the most recent in vitro study on ocular isolates [11]. The ratios for each strain are reported in Table 4. The actual concentrations of levofloxacin in aqueous humour abundantly exceeded the MICs in the majority of the in vitro–tested strains, with the only exception of MSSA and of some *Enterococcus* spp.

**Table 4:** Ratio between aqueous humour concentrations in patients after ocular instillation of L-DSP (levofloxacin + dexamethasone sodium phosphate) and in vitro MICs of levofloxacin on ocular isolates (after Yamaguchi et al. 2012 ^25^, modified)

Bacterial strain	No. isolates	MIC90 (μg/l)	AH concentrations /MIC90 ratio LL–UL 95% CI
*Streptococcus pyogenes*	434	2.0	297–414
*Streptococcus pneumoniae*	661	1.0	596–828
*Methicillin-susceptible Staphylococcus aureus*	745	0.5	1191–1657
MSSA: Methicillin-susceptible coagulase-negative *Staphylococci*	557	4.0	149–207
MRSA: Methicillin-resistant *Staphylococcus aureus*	719	> 64	< 9.3–< 12.9
Methicillin-resistant coagulase-negative *Staphylococci*	732	16.0	37.2–51.8
*Enterococcus faecalis*	641	32	18.6–25.88
*Enterococcus faecium*	591	64	9.3–12.9
*Moraxella catarrhalis*	566	0.06	9926–13,804
*Neisseria gonorrhoeae*	80	16	37.2–51.8
*Escherichia coli*	741	16	37.2–51.8
*Klebsiella pneumoniae*	678	0.5	1191–1657
*Citrobacter* spp.	603	1.0	596–828
*Enterobacter* spp.	657	0.5	1191–1657
*Proteus mirabilis*	590	8.0	74.44–104
*Indole-positive Proteus* spp.	521	0.5	1191–1657
*Serratia marcescens*	650	2.0	297–414
*Salmonella* spp.	194	0.125	4764–6626
*Haemophilus influenzae*	660	0.03	19,851–27,609
*Acinetobacter* spp.	577	4.0	149–207
*Pseudomonas aeruginosa* UTI	609	64	9.3–12.9
*Pseudomonas aeruginosa* RTI	660	8.0	74.44–104

*LL* lower limit, *UL* upper limit, *CI* confidence interval

### Safety

Only one treatment-emergent adverse event was reported: mild mydriasis in the DSP alone arm.

## Discussion

The combination of dexamethasone sodium phosphate, corresponding to dexamethasone 1 mg/ml, plus levofloxacin 5 mg/ml (L-DSP) tested in this study is the first combination of a steroid and levofloxacin under development as eye drops. The administration schedule (− 90 and − 60 min before paracentesis) was an acceptable compromise between the aim to reduce as least as possible the discomfort to the patient, before the scheduled surgery, and the need to achieve the best approximation to the C_max_ of either drug. According to previous data, the peak of levofloxacin concentrations in aqueous humour occurs 2 h after instillation, with a very small difference between levofloxacin concentrations at 60 and 120 min [12]. On the other hand, dexamethasone levels in the aqueous humour peak between 90 and 150 min after ocular instillation [4]. Repeated administrations are a common procedure in such studies [12, 13], and the choice of the two doses at − 90 and − 60 min aimed both to minimise the probability of levels under the LLOQ and to approximate the t_max_ for both analytes. The late administration of oxybuprocaine and of povidone-iodine (at least 80 and 50 min after the first and second drop, respectively) would not have affected the absorption of test or reference drugs, and the matrix effect was excluded during the validation study, as detailed in Online Resource 2.

The mean concentration of levofloxacin in L-DSP group corresponded to 711.899 ng/ml (95% CI 595.538; 828.260), and the fraction *F* of levofloxacin dose absorbed in the anterior chamber at the time of AH withdrawal was 3.8 · 10^−4^. There was a minimum difference (8% lower, not relevant) in the aqueous humour concentration of levofloxacin in the L-DSP arm compared with levofloxacin alone arm. In order to understand the clinical relevance of that result, the aqueous humour concentrations at the investigated time (supposed to correspond to the AH C_max_) have been compared with the minimum inhibitory concentrations (MIC) reported by the most recent in vitro study on ocular isolates [11]. It is evident that the actual concentrations of levofloxacin in aqueous humour abundantly exceeded the MICs in the majority of the in vitro–tested strains, with the only exception of MSSA and of some *Enterococcus* spp. The ESCRS Guidelines for Prevention and Treatment of Endophthalmitis Following Cataract Surgery [1] underline a number of studies confirming that a concentration/MIC ratio above approximately 30 (for many gram-positive strains), and above 100 (for gram-negative) was needed to eradicate bacteria. Our study confirmed that levofloxacin concentrations well above the MICs are found after ocular instillation of the test product, with AH concentration/MIC ratio exceeding the threshold of 30 in gram-positive and of 100 in gram-negative bacteria in the majority of the isolated strains.

Dexamethasone sodium phosphate concentrations in aqueous humour resulted to be under the LLOQ for all specimens. Free dexamethasone levels were measurable in almost all AH specimens, showing that the DSP was hydrolysed to the active metabolite in all samples. The aqueous humour concentrations of dexamethasone were equal to 0.030 nmol/ml (95% CI (0.025; 0.035)) in the L-DSP arm (corresponding to 11.774 ng/ml (95% CI 9.812; 13.736)) and slightly larger in the single-agent arm. The fraction *F* of dexamethasone dose absorbed was about 0.3 · 10^−4^, less than one tenth of that of levofloxacin. Nevertheless, the concentrations are great enough to carry out their expected pharmacological activity. If we consider that oral dexamethasone as an anti-inflammatory agent in ocular conditions is approved for a 2-mg daily dose [14], and that the volume of distribution at the steady state V_ss_ is about 1 l/kg in women, one half in men [15], assuming an 81% bioavailability [16] and a homogeneous distribution, then the quantity of dexamethasone reaching the anterior chamber (161.2 μl) after systemic administration would theoretically be about 0.011 nMoles, i.e. the same order of magnitude as the dexamethasone amount measured in the anterior chamber in our study. The ocular instillation of DSP is very efficient in this respect, and this was confirmed by the pivotal investigation of L-DSP in a controlled study [17] versus tobramycin and dexamethasone eye drop suspension, which was considered up to now the gold standard for topical treatment in the intended indication.

Notably, aqueous humour concentrations of dexamethasone were slightly lower in the L-DSP combination arm than in the single-agent DSP arm. Rather than the effect of a pharmacokinetic interaction between the two active ingredients of the combination product, the slightly larger penetration of dexamethasone in the AH of patients in the reference group may have been due to the higher concentration of the active ingredient in the commercial preparation. In fact, it is well-known that the most important mechanism of elimination of active ingredients from the cul-de-sac is drainage through tear fluid, which may have been more efficient in the presence of a larger volume of eye drops instilled for the test product compared with the reference drug. DSP was not detected in the samples of any of the three treatment groups, even when DSP was part of the instilled dose, most likely due to its full hydrolysis to free dexamethasone.

Since the study purpose was to evaluate the penetration of levofloxacin and dexamethasone sodium phosphate into the AH rather than to compare treatments groups, the sample size was based on the precision of the estimate, and no formal tests were performed to assess whether any differences in arms were statistically significant.

Postoperative inflammation usually is self-limiting, but steroidal drugs are often prescribed to prevent and treat postoperative inflammation, shortening recovery time and improving ocular comfort. The combined administration of a corticosteroid and an antibiotic provides a powerful anti-inflammatory effect, and at the same time a preventive and therapeutic action against infections.

Combined eye drops are comfortable for patients and improve medication compliance. This is of great importance in elderly persons, who represent at least 80% of the target population of L-DSP. Moreover, FDCs are superior to extemporaneous combinations of different products, as the FDCs are guaranteed by chemical-pharmaceutical investigation as well as by preclinical and clinical studies. Not only the efficacy and safety in humans but also the non-clinical toxicity profile and safety margin for human use are carefully investigated for FDCs. On the contrary, extemporaneous combinations are mostly based on empirical use, and their risk for patients may be underestimated. FDCs also favour the proper administration of the two agents, reduce the possibility of inaccurate dosage and improve patient compliance with medication. Finally, the use of a FDC investigated in a well-designed absorption study would avoid the reciprocate dilution in the cul-de-sac of empirically combined active ingredients and possible consequent decrease of efficacy of either product.

In conclusion, our results confirm that no interaction is evident on the corneal penetration of levofloxacin and dexamethasone, which reach pharmacologically active concentrations when instilled as fixed combination eye drops to patients undergoing cataract surgery.

## Electronic supplementary material

Online Resource 1 (DOCX 16 kb)

Online Resource 2 (DOCX 14 kb)

## Footnotes

1 Levendex® NTC srl, Milan, Italy. Brands in other countries: Ducressa®, Sevendoc®
