# Supplementary material for: Aqueous humour concentrations after topical apPlication of combinEd levofloxacin-dexamethasone eye dRops and of its single components: a randoMised, assEssor-blinded, parallel-group study in patients undergoing cataract surgery: the iPERME study
Source: Eur J Clin Pharmacol. 2020 Apr 13;76(7):929–37. doi: 10.1007/s00228-020-02863-7 (PMC7306033; doi:10.1007/s00228-020-02863-7)
Supplement: Supplementary file 1 — (DOCX 16 kb) [file 228_2020_2863_MOESM1_ESM.docx]

**Article title**

Aqueous humour concentrations after topical apPlication of combinEd levofloxacin-dexamethasone eye dRops and of its single components: a randoMized, assEssor-blinded, parallel-group study in patients undergoing cataract surgery: the iPERME study

**Journal**

Eur J Clin Pharmacol

**Authors**

Michele Figus, Chiara Posarelli, Dario Romano, Marco Nardi, Luca Rossetti

**Corresponding Author**

Michele Figus, Department of Surgical, Medical, Molecular Pathology and of Critical Area, University of Pisa, Pisa, Italy. Email: [michele.figus@unipi.it](mailto:michele.figus@unipi.it)

**Online Resource 1**

**Analytical method**

The analytes were aqueous humour (AH) concentration of levofloxacin, dexamethasone sodium phosphate (DSP) and dexamethasone. A full validation of a specific liquid chromatography tandem mass spectrometry (LC-MS/MS) method quantifying levofloxacin, DSP and its metabolite dexamethasone in human AH was performed according to a predefined study protocol.

The method consisted of internal standard (IS) addition, precipitation with methanol, then chromatographic separation under gradient conditions and MS/MS detection. The chromatographic separation was done by reverse-phase HPLC (column: Thermo Fisher HYPERSYL GOLD C 50x3 mm, 3 µm) under gradient elution mode and detection by MS/MS. Acquisition mode: Multiple Reaction Monitoring (MRM).

The method was validated to investigate matrix effect, recovery efficiency, within and between-run accuracy, within and between-run precision, linearity, lower limit of quantitation, carry-over effect, dilution integrity, short-term stability in matrix and in frozen matrix, freeze-thaw cycles stability, autosampler stability to fulfil EMA Guideline requirements on bioanalytical method validation [1] and was performed in accordance with PRC Ticinum Lab SOPs.

Because the human AH is a rare matrix and its collection is difficult (since it is only possible by anterior chamber paracentesis), as deviation from the a.m. EMA Guideline [1], method selectivity was evaluated on a single batch of artificial aqueous humour (AHA) instead of on matrix coming from six different sources. AHA was recreated and utilized as matrix during the validation study. According to Macri 2015 [2], no differences in the analysis of ophthalmological drugs were found between artificial and human AH; thus, the artificial one may be used to generate a matrix-based standard curve for method analyte quantification.

Long-term stability in matrix at -80°C was also performed. Stability of analyte stock solutions and stability of Internal Standard stock solutions are available.

Recovery efficiency and dilution integrity were also tested as part of the validation study.

**Analytical procedure**

The AH, stored in vials at -80°C, was subsequently analysed in a central laboratory. Vial labels contained the study kit number but no information regarding the drug received. The laboratory analyst was blinded to the drug administered and all samples were analysed for the concentration of three molecules (levofloxacin, dexamethasone sodium phosphate and dexamethasone) by means of a liquid chromatography tandem mass spectrometry method (LC-MS-MS) developed and validated by the Central Laboratory Ticinumlab (Novara, Italy). Ticinumlab is a laboratory working according to Good Laboratory Practice (GLP) and qualified by the Italian Health Authority for the analysis of biological samples during human pharmacokinetic studies. Quality control samples blinded to the analyst were included following advice from the German Regulatory Agency Bundesinstitut für Arzneimittel und Medizinprodukte (BfArM). To that end, the randomization list included kit numbers corresponding to quality control samples. A different analyst prepared twelve quality control (QC) samples (4 of each active principle) with synthetic AH and a known concentration of the active ingredients in pre-labelled vials at the central laboratory in the presence of personnel belonging to an independent Contract Research Organization (CRO) (OPIS srl, Desio, Italy). The twelve QC samples corresponded to 10% of the true biological samples obtained from the patients. All the samples were tested in one analytical run made up of a calibration curve, QC samples at three concentration levels, in-house control samples and study samples. QC samples and in-house control samples were randomly distributed in the analytical run. Study samples were analysed as single injection. QC samples that are NLT ±15% of the nominal value were rejected in terms of 67% of the total QC samples and 50% of QC samples per level.

The analytical procedure was blinded in full GCLP conditions.

References:

1. EMEA/CHMP/EWP/192217/2009: Guideline on bioanalytical method validation.
2. Macri A, Marini V, Sangalli G. et al. An Artificial Aqueous Humour as a Standard Matrix to Assess Drug Concentration in the Anterior Chamber by High Performance Liquid Chromatography Method, Clin.Lin. Lab. 2015;61:47-52.
