# Supplementary material for: Aqueous humour concentrations after topical apPlication of combinEd levofloxacin-dexamethasone eye dRops and of its single components: a randoMised, assEssor-blinded, parallel-group study in patients undergoing cataract surgery: the iPERME study
Source: Eur J Clin Pharmacol. 2020 Apr 13;76(7):929–37. doi: 10.1007/s00228-020-02863-7 (PMC7306033; doi:10.1007/s00228-020-02863-7)
Supplement: Supplementary file 2 — (DOCX 14 kb) [file 228_2020_2863_MOESM2_ESM.docx]

**Article title**

Aqueous humour concentrations after topical apPlication of combinEd levofloxacin-dexamethasone eye dRops and of its single components: a randoMized, assEssor-blinded, parallel-group study in patients undergoing cataract surgery: the iPERME study

**Journal**

Eur J Clin Pharmacol

**Authors**

Michele Figus, Chiara Posarelli, Dario Romano, Marco Nardi, Luca Rossetti

**Corresponding Author**

Michele Figus, Department of Surgical, Medical, Molecular Pathology and of Critical Area, University of Pisa, Pisa, Italy. Email: [michele.figus@unipi.it](mailto:michele.figus@unipi.it)

**Online Resource 2**

**Analytical method validation**

According to the validation study:

- Selectivity in blank matrix was ≤ 20% of the lower limit of quantitation (LLOQ) for the analytes and ≤ 5% for the IS.
- Retention times were about 2.6, 4.1, 4.6 and 5.7 minutes for levofloxacin, DSP, dexamethasone and IS (progesterone) respectively.
- Linearity, precision and accuracy: the method was linear in the concentration range 0.058-1.936 nmol/ml for DSP and 0.013-2.548 nmol/ml for dexamethasone with mean correlation coefficients (r) of 0.9973 and 0.9984, respectively. For DSP, precision ranged between 2% (0.097 nmol/ml) and 6% (1.743 nmol/ml); accuracy between 94% (0.097 nmol/ml) and 103% (0.058 nmol/ml). For dexamethasone, precision ranged between 2% (0.255 nmol/ml) and 7% (0.025 nmol/ml); accuracy between 95% (0.127 nmol/ml) and 104% (1.274/ml).
- The method was linear for levofloxacin aqueous humour concentrations in the range of 0.014-2.747 nmol/ml. The mean correlation coefficient (r) was 0.9993. Precision ranged between 4% (1.384 nmol/ml) and 10% (0.014 nmol/ml) while accuracy ranged between 98% (0.138 nmol/ml) and 104% (0.028 nmol/ml).
- Carry over: a carry-over effect was observed for DSP but was minimized by injecting three blanks in sequence after samples with anticipated high concentrations (QC). No carry over was observed for either levofloxacin or dexamethasone.
- LLOQ was 0.014 nmol/ml for levofloxacin, 0.058 nmol/ml for DSP and 0.013 nmol/ml for dexamethasone.
- Within and between-run accuracy and precision were demonstrated.
- Matrix effect was excluded in blank matrix spiked with analytes after extraction in matrix and in water/methanol 50/50 (v/v) at a low and at a high level of concentration. Matrix effect was also excluded in presence of oxybuprocaine, iodopovidone and benzalkonium chloride.
